# A 3D CBCT-Based Approach to Assessing Effective Radiation Penetration Depth in Intraoperative Electron Radiotherapy

**DOI:** 10.3390/diagnostics16213664

**Published:** 2026-11-05

**Authors:** Markus Stana, Klarissa Ellmauer, Sara Vockner, Ivan M. Messner, Gerd Fastner, Christoph Fussl, Brane Grambozov, Julia Kaiser, Johannes Berchtold, Andreas Sir, Ninette Scharle, Philipp Scherer, Christoph Gaisberger, Falk Röder, Matthias Mattke

**Affiliations:** 1 Department of Radiation Oncology, Paracelsus Medical University, 5020 Salzburg, Austriam.mattke@salk.at (M.M.); 2 Department of Gynecology, Paracelsus Medical University, 5020 Salzburg, Austria; 3 radART-Institute for Research and Development on Advanced Radiation Technologies, 5020 Salzburg, Austria

**Keywords:** mobile cone-beam CT, medical imaging, image guided radiotherapy, intraoperative diagnostics, IOeRT, organ at risk sparing, target coverage, precision medicine, individualized treatment delivery, breast cancer

## Abstract

**Background/Objectives**: Accurate characterization of treatment geometry is essential for intraoperative electron radiotherapy (IOeRT) treatment planning. Mobile cone-beam computed tomography (CBCT) enables volumetric assessment of intraoperative anatomy beyond conventional point-based measurements. This study compared clinically applied distance measurement techniques with geometric distance descriptors retrospectively extracted from comprehensive three-dimensional CBCT datasets. **Methods**: Ninety-six patients receiving IOeRT as a tumor-bed boost during breast-conserving surgery were included. Manual CBCT measurements (mCBCT) were compared with mean (USavg) and minimum (USmin) ultrasound measurements per patient. Comprehensive 3D distance extraction was performed in 82 CBCT datasets using an approximated thoracic-wall surface as the geometric reference. Minimum and first-quartile distances were evaluated across the complete applicator cross-section and within a 1-cm-diameter inner disk around the central beam axis. **Results**: The mean paired difference between mCBCT and USavg was 0.3 mm and was not statistically significant, whereas mCBCT measurements exceeded USmin by an average of 1.5 mm. In the principal comparison, the minimum CBCT-derived distance within the inner disk was 1.4 mm smaller than mCBCT. Across the complete applicator area, minimum distances were 9.1-10.7 mm smaller than the clinical measurements. The first-quartile distance across all surface points was 1.4 mm smaller than mCBCT, whereas the corresponding inner-disk difference was not statistically significant after correction. Isodose-atlas-based dose-volume analysis indicated that a 1.5-mm change in assumed penetration depth could correspond to an approximately 7% change in the 90% isodose volume at shallow depths. **Conclusions**: Clinical CBCT and ultrasound measurements showed small group-level differences but substantial patient-level variability. Comprehensive 3D CBCT analysis revealed spatial variations in tissue depth not fully represented by the clinical measurements, highlighting the limitations of characterizing treatment geometry using a single distance. Although anatomical accuracy and patient-specific dosimetric consequences remain to be established, these findings support the potential of intraoperative 3D CBCT to advance IOeRT toward individualized, image-guided treatment planning.

## 1. Introduction

Treatment planning in intraoperative electron radiation therapy (IOeRT) is challenging despite its comparatively simple parameter space. Unlike most modern radiotherapy techniques, which rely on inverse planning using segmented voxel-based 3D patient models, IOeRT planning is still largely based on look-up tables and empirical dose data [1,2]. Approaches to introduce 3D treatment planning were proposed more than a decade ago [3,4], yet the need for a dedicated clinical planning system remains largely unmet, partly because of the strict time constraints in the operating room [5]. Clinical trials exploring this long-sought methodology have been initiated only recently [6].

Current IOeRT dose planning assumes that dose distributions measured in a simplified water phantom geometry adequately represent those in patient tissue, forming the basis of an isodose atlas [7]. While specialized solutions such as Prelude^®^ IORT treatment planning software (MedCom Gesellschaft für Medizinische Bildverarbeitung mbH, Darmstadt, Germany) and the open-source package KALI [8] can support this process, treatment planning remains essentially a selection of the most appropriate isodose distribution based on patient-specific geometric parameters.

Apart from applicator positioning, four parameters define the dose distribution. The first is the prescribed dose, typically normalized either to the point of maximum dose on the central beam axis or to the corresponding 90% isodose line [9]. Dose escalation studies have further refined prescription strategies, particularly in breast cancer treatment [10]. The second and third parameters are the applicator diameter and bevel angle, which determine the lateral extent of the treated volume and are selected jointly by the surgeon and radiation oncologist based on tumor characteristics (e.g., tumor size, biology, uni-/multifocality) and the surgical approach (e.g., the extent of clear resection margins and the dimensions/geometry of the defined tumor bed). The fourth parameter is the beam energy determining the effective radiation penetration depth, which must provide sufficient target coverage while respecting the proximity of organs at risk (OARs), e.g., ribs and thoracic wall.

Although determining effective radiation penetration depth appears straightforward, no consensus or formal recommendation exists regarding its assessment. Principles for the respective target volume treated by IOeRT, either as an anticipated boost or as single full-dose partial breast irradiation (PBI), are substantially based on pathological data reported by Holland [11] and were elaborated in detail by Fastner [9]. In short, after tumor excision during breast-conserving surgery (BCS), the surgeon approximates the resection margins toward a center using temporary sutures to form the tumor bed. Taking into consideration the extent of clear resection margins of at least 30 mm in all directions from the macroscopic tumor edge, a volume is defined, which should be encompassed by 90% of the prescribed dose in order to capture 90% of subclinical invasive tumor cells. The depth of the breast tissue to be irradiated can be assessed intraoperatively by ultrasound (US) or by inserting a needle probe at representative locations, depending on the shape of the tumor bed and chest wall curvature [9]. When a protective shielding plate is used, selecting the appropriate electron energy is relatively simple, as the penetration depth can be chosen to cover the maximum distance between the tissue surface and the shield. Without a protective plate, combining two energy levels may be necessary in order to achieve the desired dose penetration depth and meet specified dose constraints for adjacent OARs.

Preoperative imaging can provide valuable anatomical information. But tumor resection and tissue repositioning often substantially alter the anatomy during surgery. Consequently, intraoperative measurements performed with the applicator in place are required to accurately characterize treatment geometry. The needle probe technique is widely used [12] because it is simple and effective for measuring distances to dense anatomical structures such as ribs. However, it is invasive, evaluates only a single point at a time, and may overestimate distances because measurements are not always obtained parallel to the beam axis. US is non-invasive and provides two-dimensional images that allow multiple measurements within a single scan. Specialized split applicator tubes facilitate probe placement and handling, although care must be taken to avoid tissue compression, which may lead to underestimation of distances. The applicability of US is, however, somewhat limited by the geometry of the applicator tube, which restricts access to peripheral regions and precludes comprehensive assessment of the entire volume. In addition, small surface irregularities may be smoothed by the pressure applied during image acquisition, and measured distances can differ from their orthogonal projection onto the reference axis. Other techniques have been proposed and demonstrated experimentally [13], but their broader clinical applicability remains unclear. Earlier attempts to use CT imaging were either limited to preoperative geometries or required substantial technical effort [14,15,16].

The recent introduction of a mobile cone-beam CT system for in-room use (IRm: ImagingRing-mobile™ IRm, medPhoton, Salzburg, Austria; also available as Loop-X™ by BrainLAB AG, Feldkirchen, Germany) has enabled practical acquisition of intraoperative 3D patient models. Beyond providing detailed information on tumor-bed topography and applicator positioning [17], mobile CBCT enables volumetric assessment of patient-specific treatment geometry. Karius et al. [18] reported geometric instabilities and double-contouring artifacts in the mobile CBCT system. Subsequently, Messner et al. [19] investigated focal-spot position shifts and thermal drift as sources of geometric artifacts and demonstrated that corresponding geometric corrections can mitigate these effects.

In summary, while the IOeRT planning parameter space—defined by dose, applicator diameter, bevel angle, and energy—is relatively simple, identifying the optimal treatment configuration remains challenging. To investigate this issue, we present descriptive and comparative analyses of depth measurements obtained using US, needle probes, and manual measurements on CBCT images in 96 female patients treated at our institution with BCS and a planned IOeRT as a tumor-bed boost [20,21]. These results were compared with statistical parameters derived from a retrospective analysis of complete 3D CBCT datasets. Additionally, implications of differences in effective radiation penetration depth for dose planning are discussed.

To our knowledge, this is the first quantitative comparison of depth assessment techniques and the first report describing depth statistics derived from 3D datasets in an IOeRT setting.

## 2. Materials and Methods

### 2.1. Patients

Between May 2022 and April 2024, 96 patients received an IOeRT tumor-bed boost for partial breast irradiation using the Mobetron^®^ intraoperative electron linear accelerator (IntraOp Medical, Sunnyvale, CA, USA), with intraoperative CBCT and US imaging. Of the 96 patients, 47 (49.0%) were treated on the right breast and 49 (51.0%) on the left breast. A bolus was used in eight cases to ensure the minimum required effective radiation penetration depth, allowing safe application of the lowest electron energy (6 MeV) to the chest wall without shielding. All patients were treated with cylindrical applicators with a 0° bevel angle. One patient was treated using a 5-cm-diameter tube, 63 patients with a 6-cm tube, 29 with a 7-cm tube, and three patients with an 8-cm-diameter tube.

### 2.2. Target Volume and Organs at Risk

The prescribed dose was 10 Gy to the 90% isodose, normalized to the maximum dose on the central beam axis [21]. According to our institutional standard, the dose to the ribs should not exceed 5 Gy. The depth of the 45% isodose on the central beam axis (*r*_45%_), corresponding to 5 Gy under this prescription, was therefore used to determine the required effective radiation penetration depth. The Mobetron system provides three electron energies (6, 9, and 12 MeV), each associated with a specific *r*_45%_ value for different applicator diameters. Intermediate penetration depths can be achieved in 1-mm increments by combining two electron energies with appropriately weighted monitor units. The volume encompassed by the 90% isodose (*V*_90%_) was used to characterize the corresponding target-volume coverage in the reference dose distributions.

### 2.3. Clinically Applied Distance Measurement Techniques

After positioning the appropriate tube applicator over the tumor bed, several techniques were used to determine the distance between the tumor bed surface and the chest wall and thereby estimate the effective radiation penetration depth. For each patient, multiple US measurements and a cone-beam computed tomography (CBCT) scan were acquired, while additional measurements using a needle probe were performed in selected cases.

Since the standard tube applicators for the Mobetron are 30 cm long, a special tube set was used that can be split in the middle. The upper part can be removed, while the lower part is fixed to the operating table via a table clamp, which secures the target tissue while considering individual anatomical prerequisites during radiation treatment. This allowed the physician to access the tissue surface for needle-probe measurements or to apply a transducer for US measurement. Furthermore, the tubes are made of radiolucent plastic (polyoxymethylene copolymer, POM-C) rather than aluminum to enhance CBCT image quality with the applicator in place by reducing beam-hardening effects along the tube body [17].

#### 2.3.1. Ultrasound Measurements

**Figure 1:**
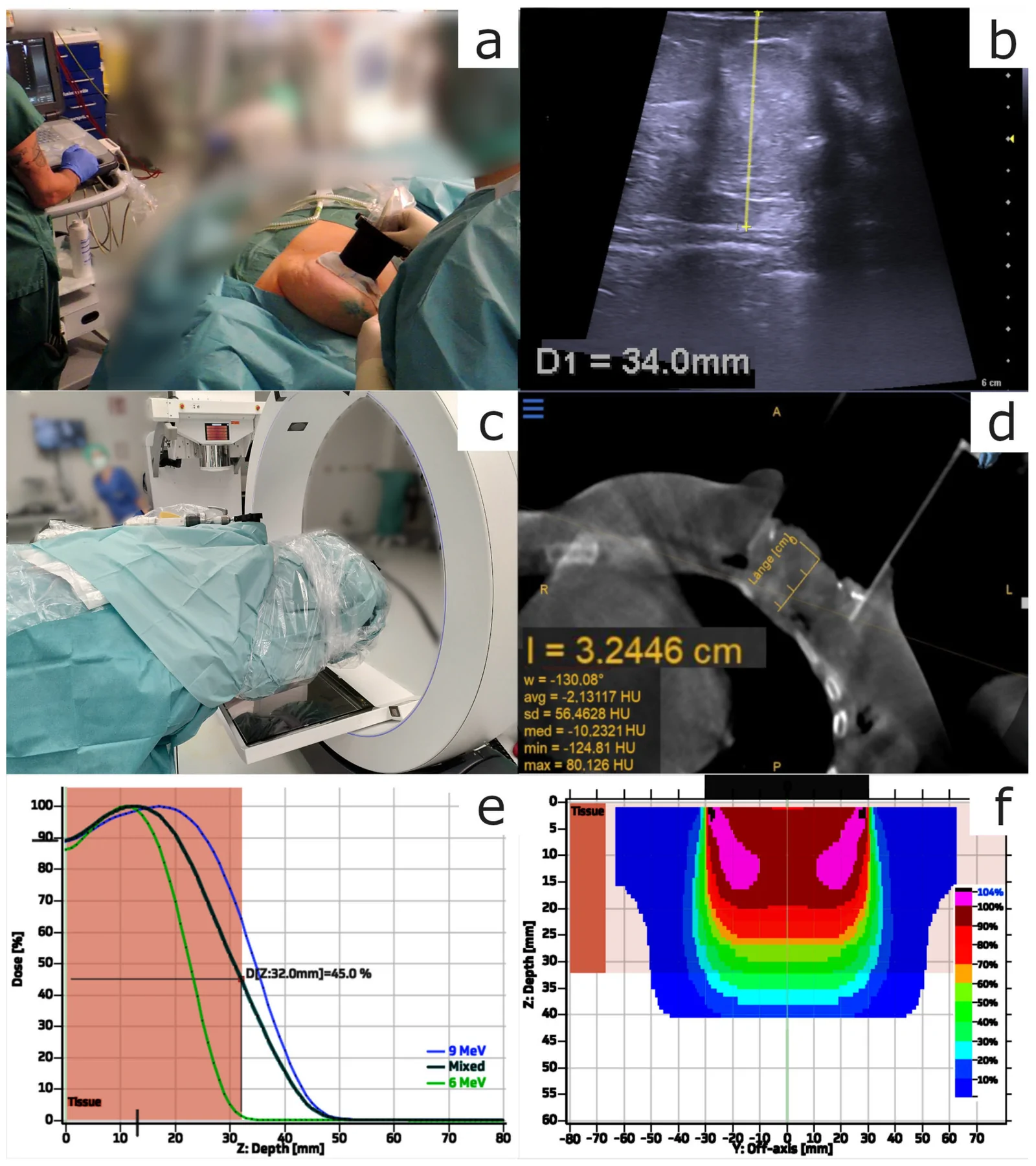
Clinical workflow: US images were recorded using a Siemens Acuson P500™ mobile US system (Siemens Healthineers, Erlangen, Germany) in combination with detachable applicator tubes to obtain the tissue surface while considering individual anatomical prerequisites during radiation treatment (**a**). A ruler tool was used to measure the distance to the thoracic wall (**b**). Afterwards, the patient was covered with sterile drapes, and the mobile cone-beam CT system (IRm: ImagingRing-mobile™ IRm, medPhoton, Salzburg, Austria; also available as Loop-X™ by BrainLAB AG, Feldkirchen, Germany) was positioned over the radiolucent patient table for imaging (**c**). After applicator alignment relative to the pleural surface, distance measurements using the integrated ruler tool were performed (**d**). The measured tissue thickness was used for treatment planning in Prelude^®^ software (MedCom Gesellschaft für Medizinische Bildverarbeitung mbH, Darmstadt, Germany) to determine the required energy (combination) (**e**). The corresponding dose profile in water is shown in (**f**).

US was the standard method for determining the distance between the tumor-bed surface and the adjacent ribs or pleura as organs at risk (OARs). A sterile US transducer was positioned within the lower section of the detachable applicator tube to acquire images directly above the tumor bed (Figure 1a). As US gel could not be applied directly to the tissue, care was taken to apply only the minimum transducer pressure necessary to avoid air gaps, since excessive pressure could lead to underestimation of the measured distances. Distances from the tumor-bed surface to the anterior edge of the ribs or pleura were measured using the digital ruler integrated into the Siemens Acuson P500™ mobile US system (Siemens Healthineers, Erlangen, Germany). The transducer position and orientation were selected by the treating radiation oncologist according to clinical judgment, occasionally including a 90° rotation to obtain an orthogonal image. For each image, the radiation oncologist selected one measurement location on the rib or pleura and measured the distance to the tumor-bed surface, as illustrated in Figure 1b. One distance measurement was recorded per image, with a mean of 2.2 measurements per patient (range: 1–4). All individual measurements were retained, and their patient-level mean and minimum were calculated as *d_USavg_* and *d_USmin_*, respectively.

#### 2.3.2. Cone-Beam Computed Tomography Measurement

After ultrasound measurements, sterile drapes were placed around the applicator, which remained uncovered during CBCT acquisition. Except for the applicator removal and repositioning required for bolus placement, no deliberate changes to the applicator position or tumor-bed geometry were made.

The patients’ extremities as well as surgical and anesthesia equipment were aligned as close as possible to the table’s central axis. This allowed the IRm to be moved over the radiolucent patient table (Figure 1c). At the start of the imaging procedure, two orthogonal X-ray projections were acquired. Based on these images, an elliptical cylindrical field of view encompassing the applicator, the underlying tissue, and a margin beyond the thoracic wall was defined for the subsequent CBCT acquisition. The reconstructed CBCT dataset was then aligned with the applicator’s central axis using multiplanar reconstruction (see, e.g., ref. [22]) to minimize geometric measurement errors.

One CBCT scan was acquired per patient. The reconstructed CBCT volume was reviewed by the radiation oncologist to assess applicator alignment relative to the pleural surface and evaluate tumor-bed flatness. Several distances between the tumor-bed surface and the underlying thoracic wall or ribs were measured at different locations using the integrated ruler tool (Figure 1d). Only the shortest measured distance was recorded as the clinical manual CBCT measurement (*d_mCBCT_*) and used to determine the effective radiation penetration depth for treatment planning (Figure 1e) and to find the corresponding dose profile in water (Figure 1f).

While all CBCT slices were reviewed, the time constraints of the intraoperative workflow precluded a comprehensive quantitative analysis of all measurable distances within the volume. Because US and CBCT images were not spatially registered, anatomical correspondence between individual measurement locations could not be established.

In the eight patients requiring a bolus, US measurements were performed before bolus placement. The applicator was subsequently removed to insert the bolus (5 or 10 mm thickness) and then repositioned before CBCT acquisition. The measured CBCT distance extended from the bolus-air interface to the underlying ribs or pleura and therefore included the bolus thickness. For treatment planning and statistical analysis, the nominal bolus thickness was subtracted from the measured distance to obtain the corresponding native tissue distance.

#### 2.3.3. Needle Probe Measurements

A sterile needle was inserted into the tumor bed until it reached the ribs. The insertion depth was marked on the needle, which was subsequently removed, and the distance between the needle tip and the mark was measured using a ruler.

This technique served as a backup when the treating radiation oncologist considered the US measurement potentially unreliable, for example, because of transducer pressure or limited image quality. Needle-probe measurements were performed at the clinician’s discretion, without predefined selection criteria, and could be repeated when necessary to obtain a clinically acceptable reading. They were performed in five patients and included only in the descriptive evaluation, not in the formal statistical comparisons. The exact number of individual readings, including repetitions, was not documented.

### 2.4. Comprehensive Extraction of Distance Information from 3D CBCT Data

Even though a comprehensive review of the entire 3D CBCT dataset was not feasible during treatment, all geometric distance information contained within the dataset could be extracted retrospectively and analyzed quantitatively.

To identify candidate points for distance measurements, relevant structures, including the tissue surface within the applicator, the applicator wall, and the thoracic wall or ribs, were segmented in the CBCT volumes. Segmentation was supported by a self-attention U-Net-based- algorithm [23], and all contours were subsequently reviewed and manually corrected where necessary. The segmented structures were analyzed using a custom script implemented in Python 3.14.0 (Python Software Foundation, Beaverton, OR, USA, 2025). Distances were determined by calculating vectors between tissue-surface points and reference points, which were considered only if they were located within an appropriate radial distance from the beam axis to reduce computational complexity. To minimize projection-related errors, only vectors aligned within 5° of the central beam axis were retained, limiting geometric deviations to below 0.5%. For each surface point, only the shortest valid distance was considered for further statistical analysis. For patients treated with a bolus, the nominal bolus thickness was also subtracted from the retrospectively extracted CBCT distances, ensuring that the analyzed distances represented native tissue thickness rather than the combined thickness of tissue and bolus.

As only applicators with a bevel angle of 0° were used, the axis of the best-fit cylinder describing the applicator was assumed to represent the central beam axis. The applicator wall was identified from the segmented structure, and a three-dimensional least-squares cylinder fit was performed to determine its orientation.

Tissue-surface points within the applicator were classified according to their radial distance from the central beam axis. A radial grid with 5 mm resolution was used, resulting in concentric annular regions extending from the beam center to the applicator edge for applicator diameters ranging from 5 to 8 cm. Surface tissue voxels within the tube-shaped applicator were selected according to the diameter and orientation of the fitted cylinder and categorized with respect to their distance from the central beam. Points within a disk with a diameter of 1 cm around the central beam corresponded to the first category (inner disk). The second category comprised points corresponding to diameters of 1–2 cm, the third annulus ranged from 2–3 cm and so on until the edge of the applicator was reached.

Direct delineation of the ribs was challenging because of limited image contrast and substantial variations in Hounsfield units (HU) between anatomical regions, such as costal cartilage and osseous rib structures. In addition, CBCT images exhibit spatially varying HU across the field of view [24], further complicating segmentation based on CT numbers. Therefore, for each patient, the outer lung boundary, corresponding to the inner thoracic wall, was expanded by a patient-specific margin to approximate the proximal rib surface in the region beneath the applicator. Candidate offsets were assessed in successive 1-mm increments, with visual inspection of all CT slices corresponding to the volume beneath the applicator. The selected margin was intended to provide the closest visual approximation of the rib boundary across the relevant region. The selected margins ranged from 5 to 10 mm (mean = 6.3 mm, standard deviation = 1.0 mm). As a sensitivity analysis, the distance descriptors and their differences from the clinical measurements were recalculated using a fixed 10-mm expansion margin for all patients.

### 2.5. Data Evaluation

For manual CBCT measurements (mCBCT), the minimum distance identified using the ruler tool on 3D-CBCT slices was recorded (*d_mCBCT_*). For US, the minimum measured distance per examination (*d_USmin_*) was extracted analogously. The mean of all available US measurements per patient (*d_USavg_*) was also included in the analysis. Needle probe measurements, due to their small number, were considered only in descriptive statistics and not used as a reference for comparative analysis.

Pairwise differences between the clinical measurement techniques were calculated on a per-patient basis. The differences were defined as Δ*_mCBCT−USavg_* = *d_mCBCT_ − d_USavg_* and Δ*_mCBCT−USmin_ = d_mCBCT_ − d_USmin_*, quantifying the deviation of the manual CBCT measurement from the mean and minimum US measurements, respectively.

Clinical mCBCT and US measurements represent local spot measurements and therefore do not capture the full spatial distribution of distances within the applicator. To characterize this distribution, distances were extracted retrospectively from the 3D CBCT datasets using the extended thoracic wall as the geometric reference. The resulting distance distributions were evaluated across spatial categories defined relative to the central beam axis (Section 2.4), including the inner disk, concentric applicator-centered rings, and the complete set of surface points within the applicator (“all points”).

For each patient and spatial category, the minimum, maximum, mean, median, and quartiles of the extracted distances were calculated. The number and spatial distribution of points varied between categories according to the applicator geometry and the spatial extent of each region. Consequently, each patient had a separate value for each statistical descriptor and category.

Because short distances to organs at risk are relevant to worst-case geometric considerations in IOeRT, the minimum extracted distance was evaluated as a geometric descriptor of the shortest distance and compared with the clinical measurements. The minimum distance within the inner disk, which represents the region surrounding the central beam axis, was selected for the principal comparison with the clinical manual CBCT measurement. The minimum distance across all surface points was additionally evaluated to characterize the shortest geometric distance anywhere beneath the applicator. However, neither minimum-distance descriptor directly determines the maximum organ-at-risk dose, typically used to define dose constraints in IOeRT [25]. This also depends on the lateral beam profile, electron energy, applicator geometry, and tissue heterogeneity. The first quartile (25%, q1) was evaluated as a complementary descriptor of the lower portion of the distance distribution because it is less sensitive than the minimum to isolated extreme values potentially arising from voxel-level segmentation or contouring errors. q1 was not treated as an independently validated anatomical reference. Absolute differences between clinical measurements and each descriptor were evaluated to compare the magnitude of the deviations irrespective of their direction. The minimum and first-quartile distances were considered complementary geometric descriptors rather than independently validated reference values.

To investigate whether the differences between measurement methods were associated with measurement magnitude, Pearson correlations were used. This analysis was performed for the clinical measurements and the CBCT-derived reference descriptors across the spatial categories.

### 2.6. Statistics

Box plots were used to visualize the distributions of the clinical measurements and the statistical descriptors derived from the comprehensive 3D CBCT distance extraction.

Normality of the paired differences was assessed using the Shapiro-Wilk test. Depending on the normality assessment, paired *t*-tests or Wilcoxon signed-rank tests were used. For all paired comparisons, 95% confidence intervals (CIs) for the mean paired differences were calculated using the t-distribution, independently of the statistical test used. For comparisons evaluated using the Wilcoxon signed-rank test, *p*-values were obtained from the Wilcoxon test, whereas the reported CIs describe the mean paired difference and were calculated separately.

The comparison between the minimum CBCT-derived distance within the inner disk and the manual CBCT measurement was considered the principal comparison because the inner disk most closely represents the region assessed during clinical distance measurement. All 12 signed-distance comparisons, including the principal comparison, were included in a single family for multiplicity adjustment using the Holm-Bonferroni method. The eight paired absolute-deviation comparisons were adjusted together as a separate family. For the two clinical comparisons of mCBCT with USavg and USmin, Holm-Bonferroni correction was applied across both tests.

Agreement between clinical measurements was assessed using Bland-Altman analysis. The mean difference (bias) and 95% limits of agreement (LoA), defined as the bias ± 1.96 times the standard deviation of the paired differences, were calculated. Approximate 95% CIs for the bias and LoA were calculated using t-based methods. Bland-Altman plots displayed the paired differences against the corresponding means of the measurements. Pearson correlation coefficients were calculated between the differences and their corresponding means as an exploratory assessment of potential proportional bias. The same difference-versus-mean approach was used to assess the relationships in the comparisons of the clinical measurements with the retrospectively derived CBCT distance descriptors. Holm-Bonferroni correction was applied across the 12 corresponding correlation tests.

A two-sided *p*-value < 0.05 was considered statistically significant. For analyses subject to multiplicity correction, significance was assessed using the adjusted *p*-values.

Statistical analyses were performed using Python (SciPy [26], pandas [27]) and Microsoft Excel [28]. Gnuplot [29] was used to create figures.

## 3. Results

**Figure 2:**
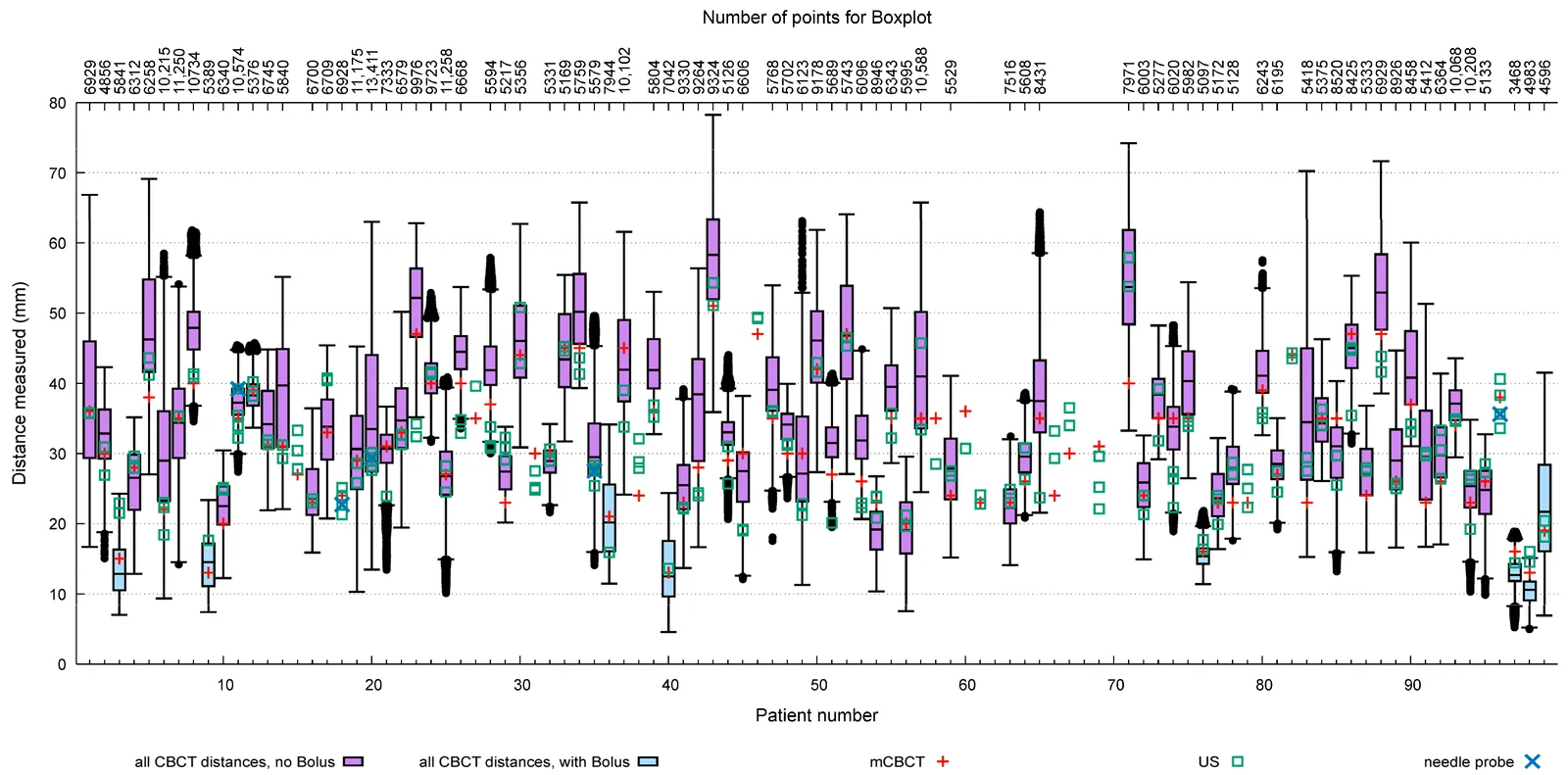
Overview of intraoperative distance measurements in 96 patients. Red crosses represent manual CBCT measurements (mCBCT), green squares represent individual ultrasound (US) distance measurements, and blue crosses indicate needle-probe measurements obtained in selected patients. Boxplots show the patient-specific distributions of retrospectively extracted 3D CBCT distances across the entire applicator-covered tissue surface in 82 patients, distinguishing cases with and without a bolus. Patient identifiers are displayed along the lower *x*-axis, while the upper *x*-axis indicates the number of extracted distance points contributing to each boxplot. The clinical measurements and CBCT-derived distributions are presented together for descriptive comparison; their anatomical measurement locations were not spatially registered.

Figure 2 provides a patient-level overview of the distance measurements obtained using the different techniques. It illustrates the variation among individual US readings within patients and the spatial distribution of retrospectively extracted 3D CBCT distances across the applicator-covered tissue surface. The clinical measurements are displayed alongside the CBCT-derived distributions for descriptive comparison, without implying anatomical correspondence between individual measurement locations. Needle-probe measurements performed in five patients (*n* = 5) are also displayed in Figure 2. Given the small, clinically selected sample and non-standardized acquisition, these measurements were evaluated descriptively and excluded from formal statistical comparisons.

In 14 of the 96 patients (14.6%), the extended thoracic-wall reference surface could not be used for comprehensive 3D distance extraction because, owing to the mediastinal position of the applicator, it did not encompass all ribs beneath the applicator. These patients remained included in the clinical measurement comparisons, while comprehensive 3D CBCT-based distance extraction was performed in the remaining 82 datasets.

A bolus was used in eight cases to match the effective radiation penetration depth to the 6 MeV electron energy profile. These cases are highlighted in a different color in Figure 2. All eight bolus cases were included in both the clinical measurement comparisons (*n* = 96) and the comprehensive retrospective 3D CBCT analysis (*n* = 82). None of the 14 patients excluded from the latter analysis had received a bolus.

### 3.1. Comparison Between Clinical Measurements

**Figure 3:**
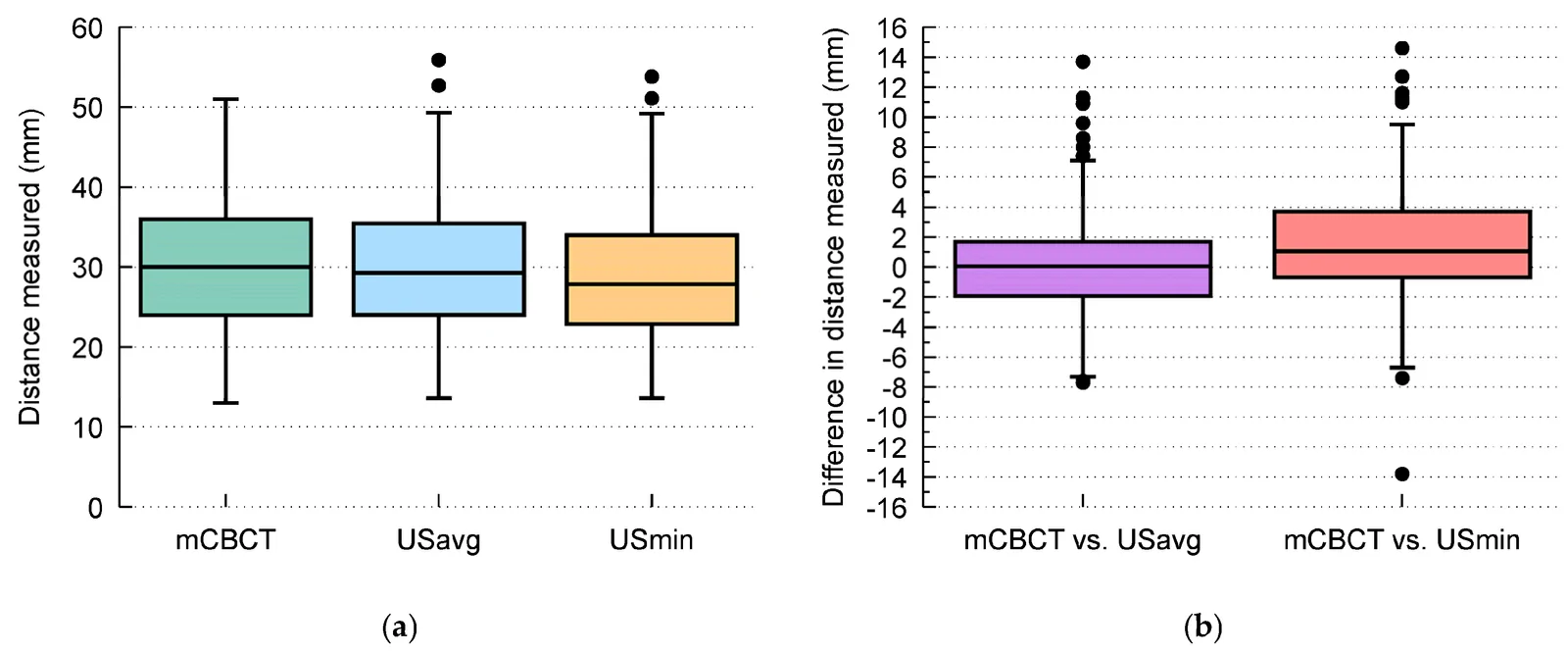
Differences between clinical distance measurements. Box plots of distances measured in 96 patients using different clinically applied methods: manual CBCT ruler measurements (mCBCT), minimum ultrasound measurements per patient (USmin), and mean ultrasound measurements per patient (USavg). (**a**) Distribution of all measured distances for each method (*d_mCBCT_*, *d_USmin_* and *d_USavg_*); (**b**) corresponding pairwise differences between methods on a per-patient basis (Δ*_mCBCT−USavg_* and Δ*_mCBCT−USmin_*).

Distance measurement results for the clinically applied techniques mCBCT, USavg and US min are represented by box plots in Figure 3a. A statistical summary for all patients and for subgroups of patients treated with and without a bolus are presented in Table 1. The patient-level differences, Δ*_mCBCT−USavg_* and Δ*_mCBCT−USmin_*, are also reported in Table 1, with corresponding box plots shown in Figure 3b. Results for the reduced dataset of 82 patients used for comprehensive extraction of distance information from 3D CBCT data were similar (see Appendix A). Although the clinical measurements and applicator-size distributions showed no pronounced descriptive differences, the anatomical exclusion criterion may still have introduced selection bias.

**Figure A1:**
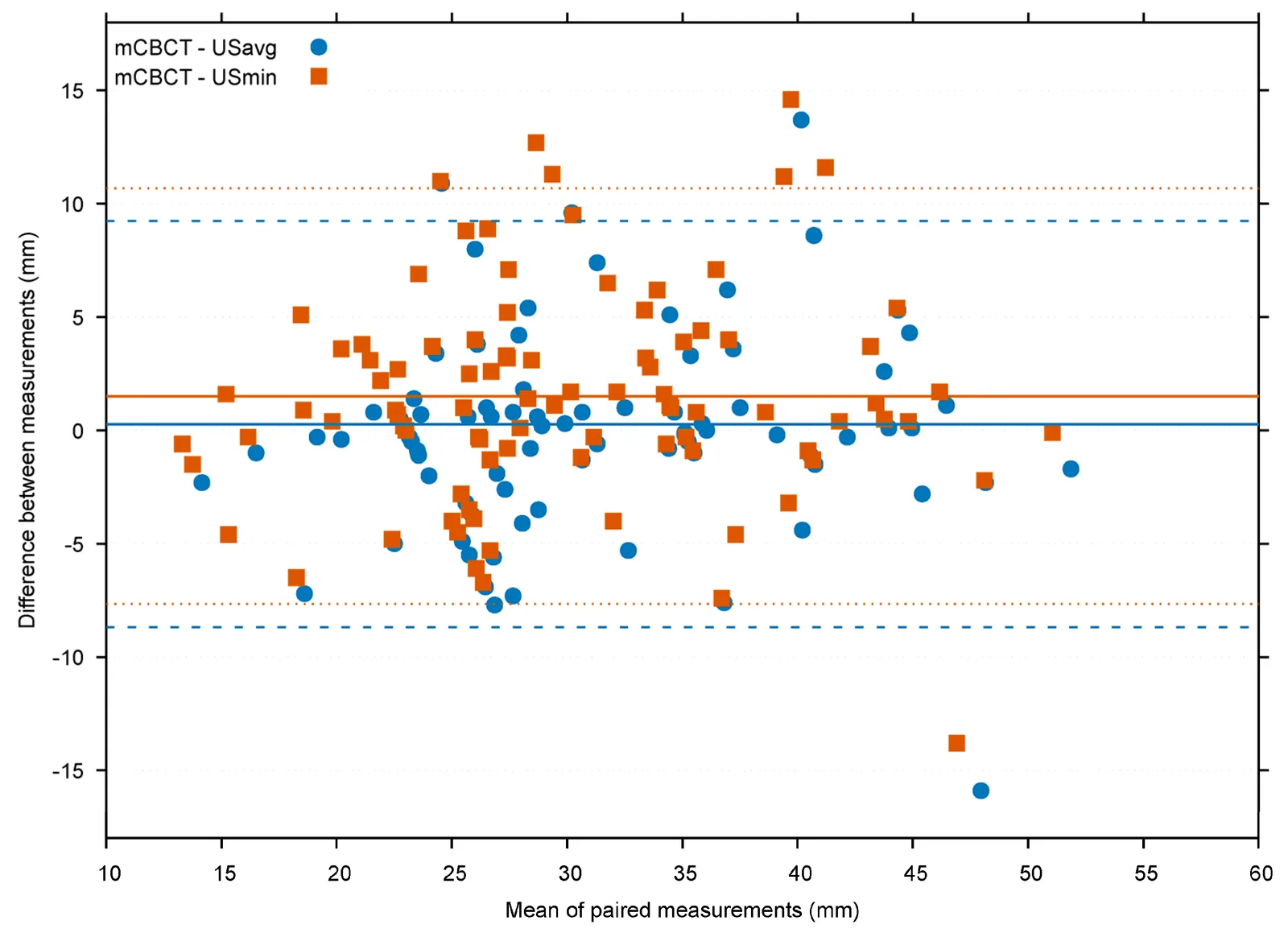
Bland-Altman plots comparing manual CBCT measurements (mCBCT) with mean ultrasound measurements (USavg, blue) and minimum ultrasound measurements (USmin, red) in 96 patients. Patient-level differences are plotted against the corresponding means of the paired measurements. Solid horizontal lines indicate the mean differences (biases), and dashed lines indicate the conventional 95% limits of agreement. The corresponding confidence intervals are reported in Section 3.1.

Paired differences were non-normally distributed for both mCBCT versus USavg (Shapiro-Wilk W = 0.960, *p* = 0.005) and mCBCT versus USmin (W = 0.971, *p* = 0.030). Accordingly, Wilcoxon signed-rank tests were used. The mean difference (bias) between mCBCT and USavg was 0.3 mm (95% CI for the mean difference: −0.7 to 1.2 mm, *p* = 0.763, Holm-adjusted *p* = 0.763), whereas *d_mCBCT_* measurements were, on average, 1.5 mm higher than *d_USmin_* measurements (95% CI: 0.6 to 2.5 mm, *p* = 0.0015, adjusted *p* = 0.0031). Agreement between the clinical measurement techniques was further assessed using Bland-Altman analysis (see also Appendix Figure A1). For mCBCT versus USavg, the conventional 95% limits of agreement were −8.7 to 9.2 mm, with 95% CIs of −10.3 to −7.1 mm for the lower limit and 7.6 to 10.8 mm for the upper limit. For mCBCT versus USmin, the corresponding limits of agreement were −7.7 to 10.7 mm, with 95% CIs of −9.3 to −6.0 mm and 9.1 to 12.3 mm, respectively. Thus, despite the small average difference between *d_mCBCT_* and *d_USavg_*, the limits of agreement indicated substantial variability in individual patient-level differences. Because both paired-difference distributions deviated from normality, the conventional limits of agreement should be interpreted cautiously.

### 3.2. Comparison of Clinical Measurements with Comprehensively Extracted Data

**Figure 4:**
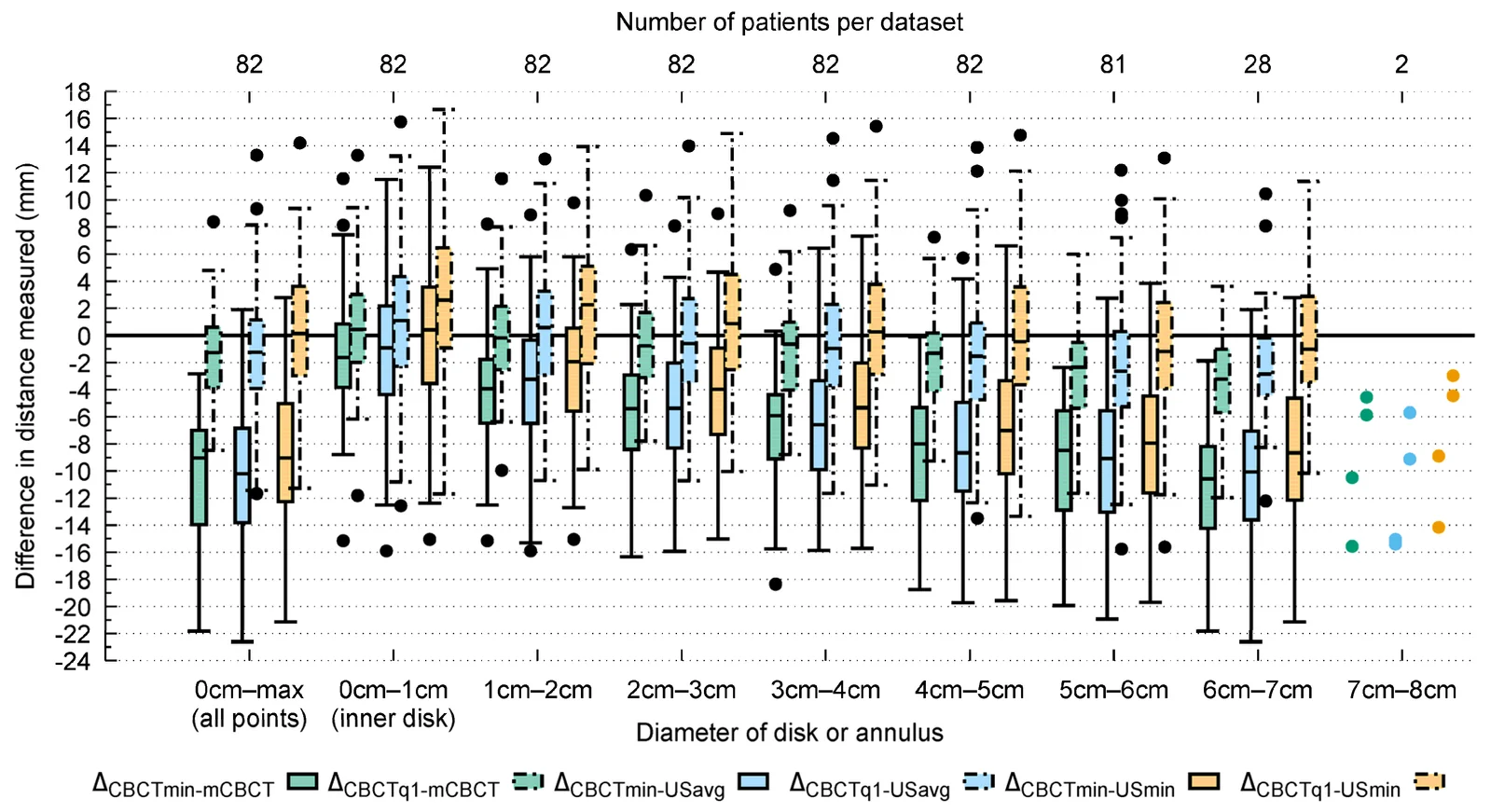
Distribution of signed differences between CBCT-derived distances and clinical measurements across spatial point categories. Differences are shown between the minimum (*d_CBCTmin_*) or first quartile (*d_CBCTq1_*) of distances obtained by comprehensive 3D CBCT extraction and manual CBCT ruler measurements (*d_mCBCT_*), average ultrasound measurements (*d_USavg_*), or minimum ultrasound measurements (*d_USmin_*). CBCT-derived distances were measured to the extended thoracic wall. Spatial categories comprise all surface points and the inner disk (diameter 0–1 cm), followed by concentric annuli at 1-cm diameter intervals up to 8 cm for the largest applicators used. All spatial categories were evaluated within the same patient cohort and therefore represent repeated within-patient analyses rather than independent patient groups. The inner disk and successive annuli are spatially distinct, whereas the all-surface-points category encompasses the entire applicator-covered tissue surface. The number of patients contributing to each category is indicated above the plot. Positive values indicate that the CBCT-derived distance exceeds the corresponding clinical measurement.

Distance analyses were performed across multiple spatial point categories, as described in Section 2. Two patient-level descriptors were evaluated from the comprehensively extracted 3D CBCT distance data: the minimum distance (*d_CBCTmin_*) and the first quartile (*d_CBCTq1_*) of the extracted distances to the extended thoracic-wall reference surface. The primary comparison was the minimum distance within the inner disk against the manually measured CBCT distance (*d_mCBCT_*), because the inner disk most closely represents the central region used for clinical measurement. Comparisons involving the first-quartile descriptor and the remaining spatial categories were considered secondary or exploratory. The distributions of the signed differences across spatial categories are shown in Figure 4, with detailed results for all surface points and the inner disk summarized in Table 2.

As discussed above, the 3D analysis was restricted to 82 patients (85.4%). For the primary comparison, the minimum CBCT-derived distance within the inner disk was, on average, 1.4 mm lower than the manually measured CBCT distance (95% CI: −2.3 to −0.5 mm, paired *t*-test *p* = 0.003, Holm-adjusted *p* = 0.019). It was also on average 1.0 mm lower than USavg (95% CI: −2.1 to 0.2 mm, paired *t*-test *p* = 0.093) and 0.2 mm higher than USmin (95% CI: −1.0 to 1.4 mm, paired *t*-test *p* = 0.711), but these results were not significant.

Across all surface points, the first-quartile descriptor was lower than the manual CBCT measurement by a mean of 1.4 mm (95% CI: −2.1 to −0.7 mm, *p* < 0.001, adjusted *p* = 0.001). The differences between the first-quartile descriptor and USavg (mean = −1.0 mm, 95% CI: −2.0 to 0.0 mm, *p* = 0.055) or USmin (mean = 0.2 mm, 95% CI: −0.8 to 1.3 mm, *p* = 0.660) were not significant after Holm correction. The minimum distance across all surface points was substantially lower than each clinical measurement: the mean differences were −10.7 mm for mCBCT, −10.3 mm for USavg, and −9.1 mm for USmin. All three comparisons remained significant after Holm correction (adjusted *p* < 0.001 for each comparison).

Within the inner disk, the first-quartile descriptor did not differ significantly from mCBCT (mean difference = 0.8 mm, 95% CI: −0.1 to 1.6 mm, *p* = 0.087) or USavg (mean difference = 1.2 mm, 95% CI: 0.0 to 2.3 mm, *p* = 0.044). Both comparisons were non-significant after Holm correction. The difference between the first-quartile descriptor and USmin was 2.4 mm (95% CI: 1.2 to 3.6 mm, *p* < 0.001, adjusted *p* = 0.001).

In conclusion, after Holm correction across all 12 signed-distance comparisons, significant differences remained for *d_CBCTq1_* versus *d_mCBCT_* across all surface points, for *d_CBCTmin_* versus all three clinical measurements across all surface points, for *d_CBCTq1_* versus *d_USmin_* within the inner disk and for the principal comparison between *d_CBCTmin_* and *d_mCBCT_* within the inner disk.

In the sensitivity analysis using a fixed 10-mm expansion margin, the mean differences between the CBCT-derived distance descriptors and the clinical measurements shifted by approximately 3.6–3.9 mm towards more negative values compared with the patient-specific margins (Appendix B, Table A3). The standard deviations remained descriptively similar, and the separation between the minimum and first-quartile descriptors was preserved: approximately 9.1–9.3 mm across all surface points and 2.1–2.2 mm within the inner disk.

For the absolute-deviation comparisons, paired differences deviated from normality in all eight comparisons (Shapiro-Wilk *p* ≤ 0.009). Wilcoxon signed-rank tests were therefore used. Across all surface points, the mean absolute deviation for the first-quartile descriptor was 2.7 mm for |Δ*_CBCTq1−mCBCT_*| and 3.6 mm for |Δ*_CBCTq1−USmin_*| (mean paired difference = −0.9 mm, *p* = 0.053). For |Δ*_CBCTq1−USavg_*|, the mean absolute deviation was 3.6 mm, with a mean paired difference of −0.9 mm against |Δ*_CBCTq1−mCBCT_*| (*p* = 0.026). Neither comparison remained significant after Holm correction. For the minimum-distance descriptor across all surface points, the mean absolute deviations were 10.7 mm for |Δ*_CBCTmin−mCBCT_*| and 9.2 mm for |Δ*_CBCTmin−USmin_*|. The mean paired difference was +1.6 mm, indicating a larger absolute deviation for mCBCT (*p* < 0.001, Holm-adjusted *p* = 0.006). The corresponding comparison with |Δ*_CBCTmin−USag_*| of 10.4 mm was not significant (*p* = 0.542).

Within the inner disk, the mean absolute deviation for the first-quartile descriptor was 3.0 mm for |Δ*_CBCTq1−mCBCT_*|, 4.7 mm for |Δ*_CBCTq1−USmin_*|, and 4.2 mm for |Δ*_CBCTq1−USavg_*|. The mean paired differences between were −1.7 mm (*p* < 0.001, Holm-adjusted *p* = 0.0006) and −1.2 mm (*p* = 0.002, Holm-adjusted *p* = 0.0111), respectively. Both comparisons remained significant after correction. For the minimum-distance descriptor within the inner disk, the mean absolute deviations were 3.4 mm for |Δ*_CBCTmin−mCBCT_*|, 4.3 mm for |Δ*_CBCTmin−USmin_*|, and 4.2 mm for |Δ*_CBCTmin−USavg_*|. Neither the comparison with USmin (mean paired difference = −0.8 mm, *p* = 0.068) nor the comparison with USavg (mean paired difference = −0.71 mm, *p* = 0.143) remained significant after Holm correction.

**Figure 5:**
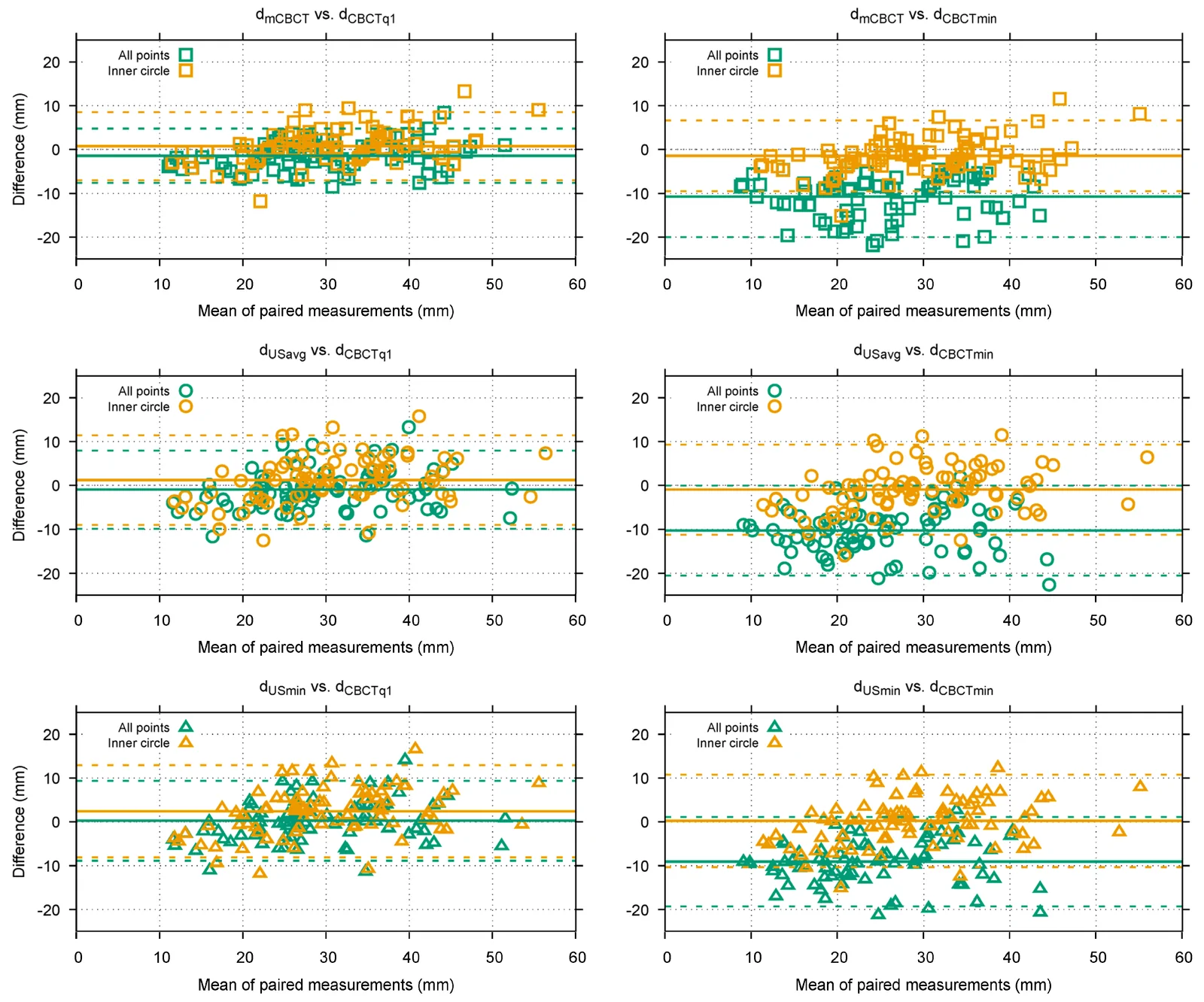
Bland-Altman plots for different point categories (all surface points and inner disk) for clinical measurement techniques referenced against statistical values from the comprehensive extraction of distance information from 3D CBCT data: first quartile (*d_CBCTq1_*, **left**) and minimum (*d_CBCTmin_*, **right**). The corresponding mean differences (bias) are represented by the solid lines, and 95% limits of agreement by broken lines.

Signed differences are shown as a function of means in Bland-Altman plots (Figure 5). In each plot, the two spatial categories (all surface points and the inner disk) are compared for clinical measurements (*d_mCBCT_*, *d_USavg_*, and *d_USmin_*) with *d_CBCTq1_* and *d_CBCTmin_*. An exploratory assessment of proportional bias examined the association between Bland-Altman differences and their corresponding means. Positive correlations were observed across all 12 comparisons (r = 0.04–0.39), with no evidence of an inverse relationship. For comparisons based on the inner disk, positive correlations were observed for all three clinical measurement methods with both the *d_CBCTq1_* (r = 0.33–0.39, Holm-adjusted *p* = 0.003–0.020) and the *d_CBCTmin_* (r = 0.31–0.36, Holm-adjusted *p* = 0.010–0.030).

Across all surface points, correlations for *d_CBCTq1_* were weaker and did not remain statistically significant after correction for multiple comparisons (r = 0.21–0.25, adjusted *p* = 0.128–0.240), while no significant correlations were found for *d_CBCTmin_* (r = 0.04–0.10, adjusted *p* = 1.000).

### 3.3. Potential Dosimetric Implications of Differences in Penetration Depth

**Figure 6:**
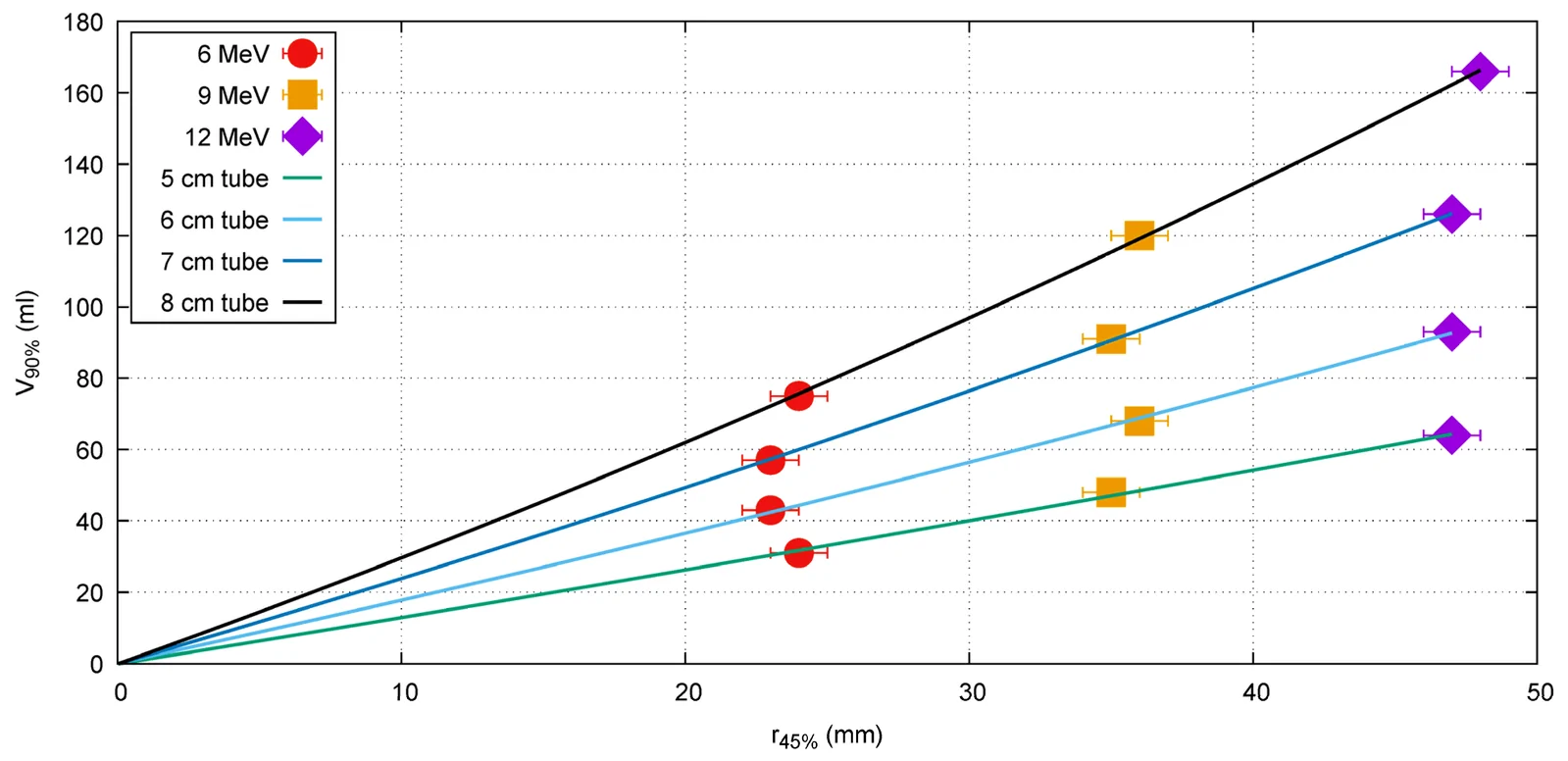
Relationship between 90% isodose volume (*V*_90%_) and 5 Gy penetration depth (*r*_45%_) for different applicator diameters. Reference values were taken from the corresponding isodose atlas for electron energies of 6, 9, and 12 MeV. Second-order polynomial fits are shown.

To evaluate the influence of changes in measured radiation penetration depth on dose distribution, volumes of the 90% isodose (*V*_90%_) calculated from the isodose atlas measured in water for different tube diameters and energies were considered. These volumes are shown as a function of 5 Gy dose depth on the central beam (*r*_45%_) in Figure 6.

The dosimetric implications of a change in *r*_45%_ depended on the operating depth. A 1.5 mm change in *r*_45%_ corresponded to a change in *V*_90%_ of approximately 6.8% to 7.2% at a shallow reference depth of about 23 mm, whereas a 1.5 mm change at a depth of about 48 mm corresponded to an increase of approximately 3.3% to 3.7%, depending on applicator diameter. These estimates represent dose-volume sensitivity rather than patient-specific 3D dose recalculation.

## 4. Discussion

In this study, we compared clinically applied methods for determining effective radiation penetration depth during IOeRT and evaluated their relationship with geometric distance descriptors derived retrospectively from comprehensive 3D CBCT datasets. The clinical measurements showed similar group-level results, but substantial differences between individual measurements. The comprehensive 3D analysis revealed additional spatial variation in the distance between the tumor-bed surface and the underlying thoracic wall, with potential implications for treatment planning.

The mean paired difference between manual CBCT measurements (*d_mCBCT_*) and mean ultrasound measurements (*d_USavg_*) was only 0.3 mm and was not statistically significant, whereas mCBCT measurements exceeded minimum ultrasound measurements (*d_USmin_*) by an average of 1.5 mm. However, Bland-Altman analysis revealed wide 95% limits of agreement (−8.7 to 9.2 mm for *d_mCBCT_* vs. *d_USavg_* and −7.7 to 10.7 mm for *d_mCBCT_* vs. *d_USmin_*), indicating substantial patient-level variability despite the small group-level differences. Thus, the techniques cannot be considered interchangeable based on the present results. Since energy mixing in our clinical workflow allows selection of penetration depths in 1-mm increments, intermethod differences may influence treatment-planning estimates, although these increments do not represent a validated measurement-error tolerance. Whether such differences would actually alter treatment selection was not investigated.

In patients treated without a bolus, mCBCT measurements tended to exceed ultrasound measurements, whereas the opposite pattern was observed in the eight patients receiving a bolus. Since ultrasound was performed before bolus placement and CBCT subsequently reflected the treatment geometry, changes in tissue position or compression may have contributed to these differences. However, the small bolus subgroup does not permit conclusions about the underlying mechanism. All eight patients treated with a bolus were included in the clinical measurement comparisons (*n* = 96) and in the retrospective 3D CBCT analysis (*n* = 82). The 14 patients excluded from the latter analysis had not received a bolus.

Additional needle-probe measurements were performed in five patients (*n* = 5). The exact number of individual readings, including repetitions, was not documented. Given the limited sample size and non-standardized acquisition, these measurements were considered descriptive only. Although useful when ultrasound measurements are considered unreliable, needle probing is invasive, and deviations from the beam-axis direction may lead to distance overestimation. Its comparative accuracy and safety could not be assessed in this study.

Retrospective extraction of distances from the 3D CBCT datasets allowed a more comprehensive characterization of the treatment geometry than individual clinical measurements. Two complementary descriptors were evaluated: the minimum distance (*d_CBCTmin_*), representing the shortest identified geometric distance, and the first quartile (*d_CBCTq1_*), characterizing the lower portion of the distance distribution while being less sensitive to isolated contouring or voxel-level errors. Their relationships with the clinical measurements depended on the descriptor and spatial region considered.

Across all surface points, *d_CBCTmin_* was, on average, 9.1–10.7 mm smaller than the clinical measurements, indicating that substantially shorter distances occurred within the complete applicator area than those represented by the clinically selected measurements. Within the 1-cm-diameter inner disk, the differences were smaller. Nevertheless, the principal comparison showed that *d_CBCTmin_* was 1.4 mm below *d_mCBCT_* on average (Holm-adjusted *p* = 0.019). This suggests that even the central region cannot necessarily be characterized by a single manual measurement. In contrast, the mean signed difference between *d_CBCTq1_* and *d_mCBCT_* was −1.4 mm across all surface points and +0.8 mm within the inner disk, illustrating the dependence of the results on the selected geometric descriptor, which is also visible in Figure 5.

The paired absolute-deviation comparisons further demonstrated a similar dependence. Relative to *d_CBCTq1_* within the inner disk, *d_mCBCT_* showed smaller absolute deviations than both *d_USmin_* (mean paired difference = −1.7 mm, Holm-adjusted *p* < 0.001) and *d_USavg_* (−1.2 mm, Holm-adjusted *p* = 0.011). However, differences were not significant for *d_CBCTq1_* across all surface points. Conversely, relative to *d_CBCTmin_* across all surface points, *d_mCBCT_* showed a larger absolute deviation than *d_USmin_* (+1.6 mm, Holm-adjusted *p* = 0.006) while comparison with *d_USmin_* yielded no significance. Neither comparison with the ultrasound measurements was significant for *d_CBCTmin_* within the inner disk. Thus, the results do not demonstrate a consistent superiority of one clinical technique across descriptors and anatomical regions. Although *d_mCBCT_* generally showed lower dispersion of reference-relative differences in central areas (see Figure 4), this reflects between-patient variability rather than measurement precision. Repeatability and interobserver reproducibility were not evaluated.

Exploratory correlations between Bland-Altman differences and their corresponding means were positive in all 12 comparisons, with significant associations after Holm correction confined to the inner-disk comparisons (r = 0.31–0.39). These findings may indicate magnitude-dependent differences, but mathematical coupling and unequal measurement variances can influence such correlations. They therefore do not establish depth-dependent measurement bias or support correction factors for individual patients.

Several methodological limitations affect the interpretation of the 3D results. Reliable segmentation of the ribs throughout the complete datasets was not feasible. Therefore, the geometric reference was approximated by expanding the outer lung boundary by patient-specific margins of 5-10 mm. Although the fixed 10-mm sensitivity analysis preserved the overall separation between the minimum and first-quartile descriptors, absolute distances depended on the expansion margin. Neither the local anatomical accuracy of this surrogate nor the reproducibility of margin selection and contouring was independently established. Furthermore, manual CBCT measurements and retrospective descriptors were derived from the same acquisition, so shared imaging uncertainties may favor their agreement. The exclusion of 14 patients (14.6%) from the comprehensive analysis because of mediastinal applicator positions may also have introduced anatomy-related selection bias. Consequently, these comparisons characterize agreement with a CBCT-derived geometric surrogate rather than independently validated anatomical accuracy.

The potential dosimetric relevance of these findings is illustrated in Figure 6. In our clinical workflow, applicator position and diameter were established before the measured penetration depth was used to select the electron-energy combination required to respect the institutional rib-dose constraint of 5 Gy. At a central-axis penetration depth (*r*_45%_) of approximately 23 mm, a 1.5-mm increase in depth corresponded to a 6.8–7.2% increase in the 90% isodose volume (*V*_90%_), depending on applicator diameter. At approximately 48 mm, a 1.5-mm decrease corresponded to a 3.3–3.7% reduction in *V*_90%_. These examples demonstrate that millimeter-scale differences in assumed penetration depth can influence dose-volume estimates, particularly at shallow depths. For reference, the tolerance typically specified for energy stability in quality assurance procedures for mobile linear accelerators is ±2 mm [30].

The substantially shorter distances identified across the complete applicator area, although suggesting a much larger difference in *V*_90%_, cannot be interpreted directly as equivalent changes in central-axis penetration depth. Peripheral regions generally receive lower doses than the central beam axis, and the relationship between geometric distance and organ-at-risk dose depends on the lateral dose profile, electron energy, applicator geometry, and tissue heterogeneity. Consequently, neither the observed 9–11 mm differences nor their theoretical direct translation into large *V*_90%_ changes establish corresponding changes in patient-specific rib dose or target coverage. The present analysis did not investigate whether alternative distance measurements would have changed energy selection or clinical outcomes. Patient-specific 3D dose calculations are therefore required to determine the dosimetric consequences of the observed geometric differences.

These findings support the potential value of spatially comprehensive intraoperative imaging for IOeRT treatment planning. Conventional correction factors can address source-to-surface distance but cannot fully account for tissue heterogeneity or spatial variations in organ-at-risk proximity. Integrating volumetric imaging with dedicated dose-calculation methods could facilitate individualized evaluation of target coverage and organ-at-risk exposure, moving IOeRT planning closer to the three-dimensional approaches established in external beam radiotherapy [3,6,31]. Although no rib-related complications were observed in this cohort, the adequacy of existing maximum-dose constraints cannot be determined from these data. Future investigations could also explore dose-volume-based organ-at-risk constraints supported by clinical follow-up.

The present methodology was evaluated in patients receiving IOeRT as a tumor-bed boost during breast-conserving surgery. Its extension to full-dose partial breast irradiation is currently limited by CBCT image degradation associated with metallic shielding plates. Applications at other anatomical sites, including the pancreas, may also be conceivable but would require site-specific validation. More broadly, although uncertainties in IOeRT planning have been discussed in relation to variable clinical outcomes [32,33], the present study cannot establish such a relationship. Nevertheless, the clinical adoption of IOeRT in breast cancer treatment, although able to reduce or eliminate irradiation of adjacent organs at risk such as skin, lung, and cardiac tissue [32], remains limited, partly due to reported outcome variability [33]. It may be cautiously hypothesized that uncertainties in treatment planning contribute to this observation, even in workflows described as technically optimized in the literature [33].

The principal contribution of this study is the quantitative characterization of intraoperative geometric variation and the identification of limitations inherent in reducing three-dimensional anatomy to a single penetration-depth measurement. Independent anatomical validation, reproducibility studies, and patient-specific dose calculations are necessary before this approach can be translated into demonstrable improvements in treatment planning.

## 5. Conclusions

In this cohort of 96 patients, manual CBCT and ultrasound measurements yielded similar results at the group level, although individual differences were substantial. Retrospective extraction of distance information from 3D CBCT datasets revealed considerable spatial variation beneath the applicator. While the first-quartile distance within the inner disk did not differ significantly from the manual CBCT measurement, the minimum distance across all surface points was approximately 9–11 mm smaller than the clinical measurements. These findings indicate that a single measurement representative of the central beam axis may not adequately characterize the shortest geometric distances to organs at risk across the entire applicator area. The dose-volume analysis further illustrated that differences in penetration depth of only a few millimeters can produce relevant changes in the 90% isodose volume, depending on applicator diameter and electron energy. However, the retrospective distances were referenced to an approximated thoracic-wall surface, and the analysis did not independently validate anatomical accuracy or determine patient-specific organ-at-risk doses. The present findings therefore support the further development of spatially comprehensive, image-guided IOeRT treatment planning, while independent anatomical validation and patient-specific dose calculations are required to establish its dosimetric benefit and clinical relevance.

## Figures and Tables

**Table 1:** Statistical summary of clinical distance measurement techniques and their differences.

Comparison Between Intraoperative Measurements							
Comparison between clinical measurements over all 96 patients							
	**Mean**	**Stdev**	**Minimum**	**Maximum**	**Quartile 1**	**Median**	**Quartile 3**
*d_mCBCT_* (mm)	30.8	8.7	13.0	51.0	24.0	30.0	36.0
*d_USavg_* (mm)	30.5	8.6	13.6	55.9	24.0	29.3	35.5
*d_USmin_* (mm)	29.3	8.5	13.6	53.8	22.9	27.9	34.0
Δ*_mCBCT−USavg_* (mm)	0.3	4.5	−15.9	13.7	−2.0	0.0	1.8
Δ*_mCBCT−USmin_* (mm)	1.5	4.7	−13.8	14.6	−0.8	1.1	3.7
Comparison between clinical measurements over 8 patients treated with a bolus							
	**Mean**	**Stdev**	**Minimum**	**Maximum**	**Quartile 1**	**Median**	**Quartile 3**
*d_mCBCT_* (mm)	15.8	2.8	13.0	21.0	13.0	15.5	18.3
*d_USavg_* (mm)	16.9	2.6	13.6	22.2	14.6	16.5	18.8
*d_USmin_* (mm)	16.5	2.4	13.6	21.5	14.4	16.1	18.0
Δ*_mCBCT−USavg_* (mm)	−1.2	3.5	−7.2	5.1	−4.0	−0.8	1.1
Δ*_mCBCT−USmin_* (mm)	−0.7	3.3	−6.5	5.1	−3.8	−0.5	1.4
Comparison between clinical measurements over 88 patients treated with no bolus							
	**Mean**	**Stdev**	**Minimum**	**Maximum**	**Quartile 1**	**Median**	**Quartile 3**
*d_mCBCT_* (mm)	32.1	7.7	20.0	51.0	26.0	31.0	36.8
*d_USavg_* (mm)	31.7	7.8	19.1	55.9	25.4	30.2	35.9
*d_USmin_* (mm)	30.4	7.8	18.4	53.8	24.5	29.0	34.9
Δ*_mCBCT−USavg_* (mm)	0.4	4.6	−15.9	13.7	−1.9	0.1	2.4
Δ*_mCBCT−USmin_* (mm)	1.7	4.7	−13.8	14.6	−0.6	1.2	3.8

Abbreviations: *d_mCBCT_* = manually measured distance on CBCT; *d_USavg_* = means of all individual ultrasound (US) distance measurements per patient; *d_USmin_* = minimum US-measured distance per patient; Δ*_mCBCT−USavg_*/Δ*_mCBCT−USmin_* = difference between the manual CBCT measurement and the mean/minimum US distance for each patient.

**Table 2:** Statistical summary differences between clinical distance measurements and distance information from complete 3D dataset.

Comparison of Intraoperative Measurements with Reference Distance Metrics from the Complete CBCT Dataset							
Patient-level signed differences between clinical measurements and 3D CBCT-derived distance descriptors (*n* = 82), evaluated across all surface points (upper section) and within the inner disk (lower section).							
	**Mean**	**Stdev**	**Minimum**	**Maximum**	**Quartile 1**	**Median**	**Quartile 3**
Δ*_CBCTmin−mCBCT_* (mm)	−10.7	4.7	−21.8	−2.8	−14.1	−9.0	−6.9
Δ*_CBCTq1−mCBCT_* (mm)	−1.4	3.2	−8.5	8.4	−3.9	−1.3	−0.7
Δ*_CBCTmin−USavg_* (mm)	−10.3	5.2	−22.6	1.9	−13.8	−10.2	−6.8
Δ*_CBCTq1−USavg_* (mm)	−1.0	4.5	−11.7	13.3	−3.9	−1.2	1.2
Δ*_CBCTmin−USmin_* (mm)	−9.1	5.2	−21.1	2.8	−12.3	−9.0	−5.0
Δ*_CBCTq1−USmin_* (mm)	0.2	4.7	−11.3	14.2	−3.0	0.1	3.6
Δ*_CBCTmin−mCBCT_* (mm)	−1.4	4.1	−15.2	11.6	−3.8	−1.6	0.9
Δ*_CBCTq1−mCBCT_* (mm)	0.8	4.0	−11.8	13.3	−2.0	0.5	3.1
Δ*_CBCTmin−USavg_* (mm)	−1.0	5.2	−15.9	11.5	−4.4	−0.9	2.2
Δ*_CBCTq1−USavg_* (mm)	1.2	5.2	−12.6	15.8	−2.3	1.1	4.5
Δ*_CBCTmin−USmin_* (mm)	0.2	5.4	−15.1	12.4	−3.6	0.4	3.7
Δ*_CBCTq1−USmin_* (mm)	2.4	5.4	−11.7	16.7	−1.0	2.6	6.5

Abbreviations: Δ*_CBCTmin−mCBCT_/*Δ*_CBCTq1−mCBCT_* = patient-specific difference between the minimum value (min)/the first quartile (q1) of all CBCT-derived distance vectors parallel to the beam axis (from the surface to the organ at risk) and the manual CBCT measurement; Δ*_CBCTmin−USavg_*/Δ*_CBCTq1−USavg_* = patient-specific difference between the CBCT-derived minimum (min)/first quartile (q1) and the mean US distance; Δ*_CBCTmin−USmin_*/Δ*_CBCTq1−USmin_* = patient-specific difference between the CBCT-derived minimum (min)/first quartile (q1) and the minimum US distance.

## Data Availability

The data that support the findings of this study are available from the corresponding author upon reasonable request.

## Abbreviations

The following abbreviations are used in this manuscript:

BCS	Breast-conserving surgery
CBCT	Cone-beam computed tomography
*d_CBCTmin_*	Minimum of 3D CBCT-derived distances within a specified spatial point category for an individual patient
*d_CBCTq1_*	First quartile (25th percentile) of 3D CBCT-derived distances within a specified spatial point category for an individual patient
*d_mCBCT_*	Minimum distance measured manually on CBCT images for an individual patient
*d_USavg_*	Mean of individual ultrasound distance measurements for an individual patient
*d_USmin_*	Minimum ultrasound distance measurement for an individual patient
EBRT	External beam radiotherapy
HU	Hounsfield unit
IOeRT	Intraoperative electron radiotherapy
IRm	ImagingRing-mobile, a mobile CBCT imaging system (ImagingRing-mobile™, medPhoton, Salzburg, Austria; also available as Loop-X™ by BrainLAB AG, Feldkirchen, Germany)
OAR	Organ at risk
PBI	Partial breast irradiation
*r*_45%_	Depth of the 45% isodose in the central beam axis
US	Ultrasound
*V*_90%_	Volume encompassed by the 90% Isodose
Δ*_mCBCT−USavg_*	Difference between *d_mCBCT_* and *d_USavg_*
Δ*_mCBCT−USmin_*	Difference between *d_mCBCT_* and *d_USmin_*
Δ*_CBCTmin−mCBCT_*	Difference between *d_CBCTmin_* and *d_mCBCT_*
Δ*_CBCTmin−USavg_*	Difference between *d_CBCTmin_* and *d_USavg_*
Δ*_CBCTmin−USmin_*	Difference between *d_CBCTmin_* and *d_USmin_*
Δ*_CBCTq1−mCBCT_*	Difference between *d_CBCTq1_* and *d_mCBCT_*
Δ*_CBCTq1−USavg_*	Difference between *d_CBCTq1_* and *d_USavg_*
Δ*_CBCTq1−USmin_*	Difference between *d_CBCTq1_* and *d_USmin_*

## Appendix A

Statistical analysis of clinical measurements for the reduced dataset of 82 patients used for comparison with 3D analysis and for 14 patients excluded from 3D analysis. Comparing values for all patients (*n* = 96) and for the subgroup used for 3D analysis yielded mean mCBCT distances of 30.6 ± 8.9 mm and 31.9 ± 7.7 mm, respectively, while mean USavg distances were 30.1 ± 8.8 mm and 32.5 ± 7.6 mm, and mean USmin distances were 28.9 ± 8.6 mm and 31.2 ± 8.2 mm. The corresponding mean differences between mCBCT and USavg were 0.4 ± 4.6 mm and −0.6 ± 4.5 mm, respectively. Applicator diameters of 5, 6, 7, and 8 cm were used in 1, 53, 26, and 2 included patients and in 0, 10, 3, and 1 excluded patient, respectively.

**Table A1:** Descriptive statistics of clinical measurements in the 82 patients included in the comprehensive 3D CBCT analysis, including the bolus and no-bolus subgroups.

Comparison Between Intraoperative Measurements							
Comparison between clinical measurements over 82 patients used for 3D analysis							
	**Mean**	**Stdev**	**Minimum**	**Maximum**	**Quartile 1**	**Median**	**Quartile 3**
*d_mCBCT_* (mm)	30.6	8.8	13.0	51.0	23.0	30.0	36.0
*d_USavg_* (mm)	30.1	8.7	13.6	55.9	23.6	28.8	35.4
*d_USmin_* (mm)	28.9	8.5	13.6	53.8	22.3	27.5	34.2
Δ*_mCBCT−USavg_* (mm)	0.4	4.6	−15.9	13.7	−1.3	0.1	1.7
Δ*_mCBCT−USmin_* (mm)	1.7	4.7	−13.8	14.6	−0.5	1.2	3.6
Comparison between clinical measurements over 8 patients treated with a bolus							
	**Mean**	**Stdev**	**Minimum**	**Maximum**	**Quartile 1**	**Median**	**Quartile 3**
*d_mCBCT_* (mm)	15.8	2.8	13.0	21.0	13.0	15.5	18.3
*d_USavg_* (mm)	16.9	2.6	13.6	22.2	14.6	16.5	18.8
*d_USmin_* (mm)	16.5	2.4	13.6	21.5	14.4	16.1	18.0
Δ*_mCBCT−USavg_* (mm)	−1.2	3.5	−7.2	5.1	−4.0	−0.8	1.1
Δ*_mCBCT−USmin_* (mm)	−0.7	3.3	−6.5	5.1	−3.8	−0.5	1.4
Comparison between clinical measurements over 74 patients treated with no bolus							
	**Mean**	**Stdev**	**Minimum**	**Maximum**	**Quartile 1**	**Median**	**Quartile 3**
*d_mCBCT_* (mm)	32.2	7.7	20.0	51.0	26.0	31.0	37.0
*d_USavg_* (mm)	31.6	7.9	19.1	55.9	25.3	30.0	35.8
*d_USmin_* (mm)	30.3	7.8	18.4	53.8	24.4	29.0	35.1
Δ*_mCBCT−USavg_* (mm)	0.6	4.6	−15.9	13.7	−1.2	0.2	2.0
Δ*_mCBCT−USmin_* (mm)	1.9	4.7	−13.8	14.6	−0.3	1.5	3.7

Abbreviations: *d_mCBCT_* = distances manually measured on CBCT data; *d_USavg_* = average distance of individual measurements; *d_USmin_* = minimum measured distances per patient; Δ_*mCBCT*−*Usavg*_ = differences between manual measurement on CBCT and average US measurement for each patient individually; Δ_*mCBCT−Usmin*_ = differences between manual measurement on CBCT and minimum US measurement for each patient individually.

**Table A2:** Comparison of the 82 included patients with the 14 excluded patients, including applicator diameters and bolus use.

Variable	Included (*n* = 82)	Excluded (*n* = 14)
Clinical measurements, Mean ± Stdev (mm)		
*d_mCBCT_*	30.6 ± 8.9	31.9 ± 7.7
*d_USavg_*	30.1 ± 8.8	32.5 ± 7.6
*d_USmin_*	28.9 ± 8.6	31.2 ± 8.2
Δ*_mCBCT−USavg_*	0.4 ± 4.6	−0.6 ± 4.5
Δ*_mCBCT−USmin_*	1.7 ± 4.7	0.8 ± 4.6
Applicator diameter *n* (%)		
5 cm	1 (1.2%)	0 (0.0%)
6 cm	53 (64.6%)	10 (71.4%)
7 cm	26 (31.7%)	3 (21.4%)
8 cm	2 (2.4%)	1 (7.1%)
Bolus use *n* (%)		
Yes	8 (9.8%)	0 (0.0%)
No	74 (90.2%)	14 (100.0%)

Note: Values are presented as mean ± standard deviation or number of patients (%). Δ*_mCBCT−USavg_* and Δ*_mCBCT−USmin_* represent paired differences between manual CBCT and ultrasound measurements. The 14 patients were excluded only from comprehensive 3D CBCT analysis; all 96 patients were included in the clinical measurement comparison.

## Appendix B

Sensitivity Analysis Using a Fixed 10-mm Thoracic-Wall Expansion.

**Table A3:** Statistical summary differences between clinical distance measurements and distance information from complete 3D dataset. Patient-level signed differences between CBCT-derived geometric distance descriptors and clinical measurements using a fixed 10-mm expansion of the outer lung boundary. Results are presented separately for all surface points (upper section) and the inner disk (lower section). Values are expressed in millimeters. Negative differences indicate that the CBCT-derived distance descriptor is smaller than the corresponding clinical measurement.

Comparison of Intraoperative Measurements with Reference Distance Metrics from the Complete CBCT Dataset							
Comparison for statistical descriptors for low distances over all points (top) and inner disc(bottom)							
	**Mean**	**Stdev**	**Minimum**	**Maximum**	**Quartile 1**	**Median**	**Quartile 3**
Δ*_CBCTmin−mCBCT_* (mm)	−14.4	4.7	−26.1	−5.8	−17.7	−13.3	−11.0
Δ*_CBCTq1−mCBCT_* (mm)	−5.2	3.3	−12.6	4.4	−7.7	−4.9	−3.1
Δ*_CBCTmin−USavg_* (mm)	−13.9	5.1	−26.7	−2.3	−17.5	−13.8	−10.7
Δ*_CBCTq1−USavg_* (mm)	−4.8	4.5	−15.7	8.9	−7.6	−5.0	−2.0
Δ*_CBCTmin−USmin_* (mm)	−12.7	5.1	−25.8	−1.4	−15.9	−12.6	−9.0
Δ*_CBCTq1−USmin_* (mm)	−3.6	4.6	−15.0	9.8	−6.8	−4.0	−0.4
Δ*_CBCTmin−mCBCT_* (mm)	−5.2	4.2	−19.9	7.5	−7.5	−5.5	−3.1
Δ*_CBCTq1−mCBCT_* (mm)	−3.1	4.1	−16.9	9.1	−5.3	−3.2	−0.8
Δ*_CBCTmin−USavg_* (mm)	−4.8	5.1	−20.7	7.3	−8.0	−4.9	−1.7
Δ*_CBCTq1−USavg_* (mm)	−2.7	5.1	−17.6	11.4	−5.8	−3.1	1.0
Δ*_CBCTmin−USmin_* (mm)	−3.6	5.2	−19.8	8.2	−6.7	−3.4	−0.3
Δ*_CBCTq1−USmin_* (mm)	−1.4	5.3	−16.8	12.3	−4.7	−1.5	2.1

Abbreviations: Δ*_CBCTmin−mCBCT_/*Δ*_CBCTq1−mCBCT_* = patient-specific difference between the minimum value (min)/the first quartile (q1) of all CBCT-derived distance vectors parallel to the beam axis (from the surface to the organ at risk) and the manual CBCT measurement; Δ*_CBCTmin−USavg_*/Δ*_CBCTq1−USavg_* = patient-specific difference between the CBCT-derived minimum (min)/first quartile (q1) and the mean US distance; Δ*_CBCTmin−USmin_*/Δ*_CBCTq1−USmin_* = patient-specific difference between the CBCT-derived minimum (min)/first quartile (q1) and the minimum US distance.

## Appendix C

Bland-Altman plots for comparing clinical measurements mCBCT against USavg and USmin. Bland-Altman Analysis of Clinical Distance Measurements.

## References

[1] Stern R.L., Heaton R., Fraser M.W., Goddu S.M., Kirby T.H., Lam K.L., Molineu A., Zhu T.C., Group A.T. (2011). Verification of monitor unit calculations for non-IMRT clinical radiotherapy: Report of AAPM Task Group 114. Med. Phys..

[2] Ezzell G.A., Galvin J.M., Low D., Palta J.R., Rosen I., Sharpe M.B., Xia P., Xiao Y., Xing L., Yu C.X. (2003). Guidance document on delivery, treatment planning, and clinical implementation of IMRT: Report of the IMRT Subcommittee of the AAPM Radiation Therapy Committee. Med. Phys..

[3] Guerra P., Udias J.M., Herranz E., Santos-Miranda J.A., Herraiz J.L., Valdivieso M.F., Rodriguez R., Calama J.A., Pascau J., Calvo F.A. (2014). Feasibility assessment of the interactive use of a Monte Carlo algorithm in treatment planning for intraoperative electron radiation therapy. Phys. Med. Biol..

[4] Pascau J., Santos Miranda J.A., Calvo F.A., Bouche A., Morillo V., Gonzalez-San Segundo C., Ferrer C., Lopez Tarjuelo J., Desco M. (2012). An innovative tool for intraoperative electron beam radiotherapy simulation and planning: Description and initial evaluation by radiation oncologists. Int. J. Radiat. Oncol. Biol. Phys..

[5] Krengli M., Kruszyna-Mochalska M.M., Pasqualetti F., Malicki J. (2025). Can Advances in Artificial Intelligence Strengthen the Role of Intraoperative Radiotherapy in the Treatment of Cancer?. Cancers.

[6] Iliaskou C., Gainey M., Kollefrath M., Kuhn S., Boronikolas V., Thomsen A.R., Ruess D.A., Grosu A.L., Baltas D. (2026). Experimental evaluation of an intraoperative-imaging based workflow for electron beam radiotherapy of pancreatic cancer using in situ dosimetry. J. Appl. Clin. Med. Phys..

[7] Hensley F.W. (2017). Present state and issues in IORT Physics. Radiat. Oncol..

[8] Ayala R., Garcia R., Ruiz G., Gomez S., Santos M., Alvarez A., Jesus Garcia M., Soza Maranon A., Udias J.M., Ibanez P. (2026). Kali MC: An Open-Source Toolkit for Intraoperative Electron Radiation Therapy Treatment Planning. Biomed. Phys. Eng. Express.

[9] Fastner G., Gaisberger C., Kaiser J., Scherer P., Ciabattoni A., Petoukhova A., Sperk E., Poortmans P., Calvo F.A., Sedlmayer F. (2020). ESTRO IORT Task Force/ACROP recommendations for intraoperative radiation therapy with electrons (IOERT) in breast cancer. Radiother. Oncol..

[10] Gatzemeier W., Orecchia R., Gatti G., Intra M., Veronesi U. (2001). Intraoperative radiotherapy (IORT) in treatment of breast carcinoma—A new therapeutic alternative within the scope of breast-saving therapy? Current status and future prospects. Report of experiences from the European Institute of Oncology (EIO), Mailand. Strahlenther. Onkol..

[11] Holland R., Veling S.H.J., Mravunac M., Hendriks J.H.C.L. (1985). Histologic multifocality of tis, T1-2 breast carcinomas implications for clinical trials of breast-conserving surgery. Cancer.

[12] Lemanski C., Azria D. (2016). Intraoperative Technique with Electrons. Short Course Breast Radiotherapy: A Comprehensive Review of Hypofractionation, Partial Breast, and Intra-Operative Irradiation.

[13] Yazdani M.R., Setayeshi S., Arabalibeik H., Akbari M.E. (2017). Design, construction and performance evaluation of the target tissue thickness measurement system in intraoperative radiotherapy for breast cancer. Nucl. Instrum. Methods Phys. Res. Sect. A Accel. Spectrometers Detect. Assoc. Equip..

[14] Trifiletti D.M., Showalter T.N., Libby B., Brenin D.R., Schroen A.T., Reardon K.A., Showalter S.L. (2015). Intraoperative breast radiation therapy with image guidance: Findings from CT images obtained in a prospective trial of intraoperative high-dose-rate brachytherapy with CT on rails. Brachytherapy.

[15] Garcia-Vazquez V., Calvo F.A., Ledesma-Carbayo M.J., Sole C.V., Calvo-Haro J., Desco M., Pascau J. (2020). Intraoperative computed tomography imaging for dose calculation in intraoperative electron radiation therapy: Initial clinical observations. PLoS ONE.

[16] Hassinger T.E., Showalter T.N., Schroen A.T., Brenin D.R., Berger A.C., Libby B., Showalter S.L. (2018). Utility of CT imaging in a novel form of high-dose-rate intraoperative breast radiation therapy. J. Med. Imaging Radiat. Oncol..

[17] Roeder F., Fastner G., Fussl C., Sedlmayer F., Stana M., Berchtold J., Jager T., Presl J., Schredl P., Emmanuel K. (2023). First clinical application of image-guided intraoperative electron radiation therapy with real time intraoperative dose calculation in recurrent rectal cancer: Technical procedure. Radiat. Oncol..

[18] Karius A., Karolczak M., Strnad V., Bert C. (2022). Technical evaluation of the cone-beam computed tomography imaging performance of a novel, mobile, gantry-based X-ray system for brachytherapy. J. Appl. Clin. Med. Phys..

[19] Messner I.M., Keuschnigg P., Stollinger B., Kraihamer M., Coste-Marin J., Huber P., Kellner D., Kreuzeder E.M., Steininger P., Deutschmann H. (2024). Investigating focal spot position drift in a mobile imaging system equipped with a monobloc-based x-ray generator. Med. Phys..

[20] Fastner G., Sedlmayer F., Merz F., Deutschmann H., Reitsamer R., Menzel C., Stierle C., Farmini A., Fischer T., Ciabattoni A. (2013). IORT with electrons as boost strategy during breast conserving therapy in limited stage breast cancer: Long term results of an ISIORT pooled analysis. Radiother. Oncol..

[21] Sedlmayer F., Reitsamer R., Wenz F., Sperk E., Fussl C., Kaiser J., Ziegler I., Zehentmayr F., Deutschmann H., Kopp P. (2017). Intraoperative radiotherapy (IORT) as boost in breast cancer. Radiat. Oncol..

[22] Siegel M.J. (2003). Multiplanar and three-dimensional multi-detector row CT of thoracic vessels and airways in the pediatric population. Radiology.

[23] Vockner S., Mattke M., Messner I.M., Gaisberger C., Zehentmayr F., Ellmauer K., Ruznic E., Karner J., Fastner G., Reitsamer R. (2025). Cone-Beam CT Segmentation for Intraoperative Electron Radiotherapy Based on U-Net Variants with Transformer and Extended LSTM Approaches. Cancers.

[24] Yadegari A., Safi Y., Shahbazi S., Yaghoutiazar S., Ghazizadeh Ahsaie M. (2023). Assessment of CBCT gray value in different regions-of-interest and fields-of-view compared to Hounsfield unit. Dentomaxillofac Radiol..

[25] Sindelar W.F., Kinsella T.J. (2003). Normal tissue tolerance to intraoperative radiotherapy. Surg. Oncol. Clin. N. Am..

[26] Virtanen P., Gommers R., Oliphant T.E., Haberland M., Reddy T., Cournapeau D., Burovski E., Peterson P., Weckesser W., Bright J. (2025). SciPy: Scientific Computing Tools for Python.

[27] McKinney W. (2025). Pandas: Python Data Analysis Library.

[28] Corporation M. (2024). Microsoft Excel.

[29] Williams T., Kelley C., Felsen M. (2025). Gnuplot.

[30] Beddar A.S., Biggs P.J., Chang S., Ezzell G.A., Faddegon B.A., Hensley F.W., Mills M.D. (2006). Intraoperative radiation therapy using mobile electron linear accelerators: Report of AAPM Radiation Therapy Committee Task Group No. 72. Med. Phys..

[31] Cavedon C., Mazzarotto R. (2022). Treatment Planning in Intraoperative Radiation Therapy (IORT): Where Should We Go?. Cancers.

[32] Willett C.G., Czito B.G., Tyler D.S. (2007). Intraoperative radiation therapy. J. Clin. Oncol..

[33] Orecchia R., Veronesi U., Maisonneuve P., Galimberti V.E., Lazzari R., Veronesi P., Jereczek-Fossa B.A., Cattani F., Sangalli C., Luini A. (2021). Intraoperative irradiation for early breast cancer (ELIOT): Long-term recurrence and survival outcomes from a single-centre, randomised, phase 3 equivalence trial. Lancet Oncol..

